# Targeting the Gut Microbiota to Relieve the Symptoms of Irritable Bowel Syndrome

**DOI:** 10.3390/pathogens10121545

**Published:** 2021-11-25

**Authors:** Tomasz Wollny, Tamara Daniluk, Ewelina Piktel, Urszula Wnorowska, Anna Bukłaha, Katarzyna Głuszek, Bonita Durnaś, Robert Bucki

**Affiliations:** 1 Holy Cross Oncology Center of Kielce, Artwińskiego 3, 25-734 Kielce, Poland; tomwollny@gmail.com; 2 Department of Medical Microbiology and Nanobiomedical Engineering, Medical University of Białystok, Mickiewicza 2c, 15-222 Białystok, Poland; tamara.daniluk@umb.edu.pl (T.D.); ewelina.piktel@wp.pl (E.P.); urszula.wnorowska@umb.edu.pl (U.W.); 3 Department of Microbiological Diagnostics and Infectious Immunology, Medical University of Białystok, Waszyngtona 15a, 15-269 Białystok, Poland; anna.buklaha@umb.edu.pl; 4 Institute of Medical Science, Collegium Medicum, Jan Kochanowski University in Kielce, 25-734 Kielce, Poland; kasia.kielce@wp.pl (K.G.); Bonita.Durnas@onkol.kielce.pl (B.D.)

**Keywords:** irritable bowel syndrome, gut microbiome, small intestinal bacterial overgrowth, probiotics, microflora transplantation

## Abstract

Irritable bowel syndrome (IBS) is a common, chronic, functional disorder with a large impact on world population. Its pathophysiology is not completely revealed; however, it is certain that dysregulation of the bidirectional communications between the central nervous system (CNS) and the gut leads to motility disturbances, visceral hypersensitivity, and altered CNS processing characterized by differences in brain structure, connectivity and functional responsiveness. Emerging evidence suggests that gut microbiota exerts a marked influence on the host during health and disease. Gut microbiome disturbances can be also important for development of IBS symptoms and its modulation efficiently contributes to the therapy. In this work, we review the current knowledge about the IBS therapy, the role of gut microbiota in pathogenesis of IBS, and we discuss that its targeting may have significant impact on the effectiveness of IBS therapy.

## 1. Introduction

Irritable bowel syndrome (IBS) is a functional disorder, characterized by abdominal pain associated with a change in stool form or frequency, that has an important influence on quality of life and social functions [1]. IBS is one of the most frequent gastrointestinal conditions, which affects around 10–15% of otherwise healthy people in Europe and in the USA [2]. It has a huge impact on government spending: the sum of annual direct and indirect costs related to IBS are around 8 billion Euro in Europe, 10 billion USD in USA and around 123 billion Yuan in China [3,4,5]. Despite the prevalence of extensive research, the pathophysiology of IBS is not fully understood. However, it is believed to be a consequence of disordered communication between the gut and the brain, leading to motility disturbances, visceral hypersensitivity, and altered CNS processing, characterized by differences in brain structure, connectivity and functional responsiveness [6]. For over a decade, there has been growing evidence of the underlying role of the bacterial composition of the gastrointestinal tract in health and disease [7,8]. Moreover, the data suggesting that alternation in gut microbiota might have a role in IBS, led to the concepts involving antibiotics use in the treatment of irritable bowel syndrome [9].

## 2. IBS diagnosis and Risk Factors

Diagnosis of IBS is made based on the Rome IV criteria (published in 2016), which were developed as a result of consensus among scientists in functional gastrointestinal disorders. Clinical findings are crucial in IBS recognition, because of the absence of abnormal radiological or endoscopic features [10]. The abdominal pain associated with an alteration in either stool form or frequency, occurring for at least 6 months is essential for the IBS diagnosis [11]. Patients are sub-grouped according to their predominant stool pattern by use of the Bristol Stool Form Scale: IBS with diarrhea, IBS with constipation, IBS with mixed stool pattern, and IBS unclassified [12]. The accurate prevalence of IBS is difficult to estimate, since a generally accepted biomarker of the disease has not been discovered so far. However, epidemiological studies conducted in many countries provided data showing that IBS occurs between 5% and 10% in most studied geographical regions [13]. 

Traditionally, IBS is understood as brain-gut regulation disorder [14]. It is believed that dysregulation of the bidirectional communications system between the gastrointestinal tract and the central nervous system, mediated by many factors, is responsible for the development of many gastrointestinal symptoms and causes marked impairments in quality of life. Moreover, changes in intestinal permeability and microbiome caused by the acute gastrointestinal infection are responsible for cytokine release due to activation of mast cells and T lymphocytes, markedly influencing neural control of the gut [15]. 

**Figure 1:**
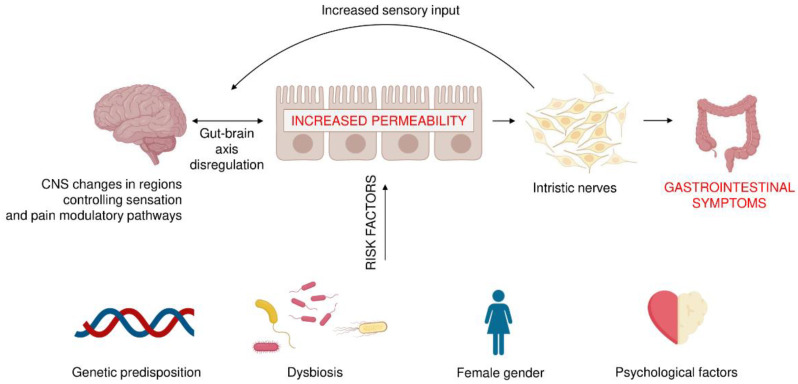
The irritable bowel syndrome: multifactorial pathogenesis and risk factors. The bacteria that remain in the gut can produce various substances that affect the nerve cells present in the gastrointestinal wall, as well as neurons in different areas of the CNS. As a result, pain levels and gastrointestinal transit can change, affecting the development of IBS. On the other hand, the spectrum of gut colonizing bacteria may depend on the genetic predisposition, diet, antibiotic treatment, gender, and the mental state of the host.

Factors that play a key role in the pathophysiology of IBS include genetic predispositions (variation of chromosome 9, mutations in sucrose-isomaltase gene and SCN5A mutation affecting smooth muscle function), present in 2% IBS patients, and physiological ones, such as abnormal motor activity, visceral hypersensitivity and disorders of the immune function of the intestinal mucosa as a result of microinflammation caused by dysbiosis, i.e., a disturbance of the composition and proportions gut microbiota, which is an essential component of gut-brain interactions. In addition, several psychological conditions, including life stress, somatization, anxiety and depression, poor social support, and abuse may be implicated with IBS (Figure 1). On the molecular level, development of IBS was reported to be associated with the disruptions in antimicrobial peptides levels, particularly defensins [16,17,18] or altered expression of secondary messengers, such as sphingosine-1-phosphate (S1P) [19,20]. Particularly, disruption of S1P was proposed as an alternative approach in the IBD (inflammatory bowel disease) treatment [19] and other inflammation-associated medical conditions [21].

IBS is diagnosed more often in women than in men; it was noticed to be lower in the group aged 50 years and older (OR: 0.75; 95% CI 0.62-0.92) than in the group younger than 50 years old [22]. While there is no confirmed data on the influence of socio-economic status on IBS, it is documented that IBS is more common in subjects with functional somatic syndromes, such as chronic fatigue and fibromyalgia [23]. Many other factors were studied (acute gastrointestinal infection, mucosal inflammation, abdominal or pelvic surgery, life stress, somatization, anxiety or depression, poor coping skills, poor social support, maladaptive cognitions or abuse), however their role is still only hypothetical. On the other hand, the frequent risk factor described is previous enteric infection, observed in about 10% of patients [24]. This condition is called post–infection IBS and arises in response to bacterial, viral or protozoal infection [25]. Authors of this meta-analysis showed that the odds of developing IBS increased by four times in exposed individuals for up to 12 months after infection (OR 4.2; 95% CI 3.1–5.7) [25]. Moreover, longer follow up of post-infection IBS patients reviled that they remained symptomatic for another 8 years [26].

## 3. Available Ways to Manage IBS 

A wide variety of symptoms and contrasting patient complaints in different types of IBS make difficult to create single algorithm for therapy. Another factor that hinders drug research, is high placebo response rate in IBS trials, reaching 30 to 40% [27]. Until recently, the first choice in IBS therapy were laxatives, antidiarrheals, and antispasmodics. However, research on the effectiveness of the majority of these preparations is uncertain, because of their suboptimal methodology and heterogeneous patient selection. Moreover, the efficacy endpoints of many trials do not meet current recommendations from the US Food and Drug Administration (FDA) or the European Medicines Agency. Additionally, some medications work only for one symptom of IBS, which does not change patients’ quality of life. For instance, polyethylene glycol was found effective in the study on 139 patients with IBS with constipation, however, no improvement in abdominal pain was noticed [28]. Research on the most commonly used antidiarrheal drug, loperamide, is sparse and covers small groups of patients [29]. Despite that, these drugs are readily used by doctors, and many patients are convinced of their effectiveness. The meta-analysis taken data from 26 trials have demonstrated that antispasmodic drugs were more effected than placebo in IBS: RR of subjects remaining symptomatic was 0.65; CI 0.56–0.76, however, side-effects were found more frequently in the drug group (RR; 1.60; CI; 1.15–2.21) [29]. Preparations, well tested and with documented effectiveness, such as otilonium, cimetropium, pinaverium, and hyoscine are not available in many countries (including Poland). It is worth noting that pinaverium studied in the Chinese population was found efficacious for abdominal pain and diarrhea [30]. Another study based on seven randomized controlled trials has proven that peppermint oil was able to reduce IBS symptoms: RR of remaining symptomatic was 0.54; CI 0.39–0.76; however, this research did not meet the requirements of the US FDA or the European Medicines Agency [29,31].

Considering that dysregulation of the gut-brain axis is crucial in the pathogenesis of IBS, the use of drugs influencing the central nervous system as a potential therapy is rational. It is believed that the use of the neuro-modulatory features of tricyclic antidepressants and their influence on slowing down gastrointestinal transit might be applied for IBS patients with abdominal pain and/or diarrhea [32]. In fact, a meta-analysis of 12 randomized controlled trials reported an RR of remaining symptomatic of 0.65 (CI 0.55–0.77) compared with placebo. However, experts emphasized that the quality of the research was low due to incorrect methodology [33]. Moreover, side effects were found more frequently with antidepressants than with placebo, which does not prompt many doctors to use them. Studies on selective serotonin reuptake inhibitors were even less encouraging; pregabalin given to 85 patients for 12 weeks in placebo-controlled trial did not result in adequate symptomatic relief [34]. 

Earlier works have investigated the effects of serotonin receptor ligands. Tegaserod, 5-HT4 agonist accelerating colon transit, was again indicated in USA (2018) for female IBS patients with constipation, who were under 65 years old and did not have existing cardiovascular disease (previously, cerebro- and cardiovascular ischemic events were observed, and the drug was withdrawn from the market [33]). Although Prucaloprid, another serotonin receptor agonist, was found to be more effective vs placebo in patients with constipation [35], its efficacy in IBS has not been confirmed in any trial, so far. An interesting observation regarding drugs acting on ion channels in red blood cells (Lubiproston, Linaclotyd, Plecanatide and Tenapanor), causing water efflux and thereby speeding up the intestinal transit, was published recently. It has been shown in placebo-controlled trials, that all these medications, by improving stool consistency, were effective in constipated IBS patients [36,37,38,39]. Unfortunately, side effects were noticed: Lubiprostone caused nausea in up to 20% of patients and other drugs were responsible for many cases of persistent diarrhea in the treatment group [40]. Knowing the psychological profile of IBS patients, it is likely to be a case of “out of the frying pan into the fire”; these preparations are not available in Poland.

The group of 5-HT3 antagonist (Alosetron, Ramosetron and Ondansetron) and peripheral opioid receptor antagonist (Eluxadoline), which slow down the gut transit and reduced visceral hypersensitivity, were investigated. They were indicated for IBS patients with diarrhea, and their efficacy over placebo was documented in stool consistency and urgency, however they more often resulted in constipation, and they did not meet essential clinical need for pain relief [40,41]. Therefore, other solutions were again taken under consideration in relation to the role of gut microbiota in IBS pathogenesis and its treatment.

## 4. The Gut Microbiome and IBS Pathogenesis

The human gut contains a collection of microbes that include commensal, symbiotic, and pathogenic bacteria, as well as fungi, viruses, archaea, and helminths, which have significant impact on the host during homeostasis and pathological conditions. It needs to be highlighted that intestinal mucus secreted by goblet cells is crucial in mediating the host-microbiota relationship, separating luminal flora from underlying epithelium [42,43,44]. It is believed that intestinal mucus is involved in the reduction of antigen exposure to the immune system, being a first line immunological barrier [45]. Moreover, it was demonstrated that some defects in GI mucus structure and functions could heighten expression of inflammatory markers influencing the host health [46]. 

A complex community of bacteria, archaea, and eukarya, estimated to consist of 10^14^ cells (that is about 10 fold greater that all human cells in human body) and located in gut, is known as the gastrointestinal tract microbiota [47]. This ecosystem is composed of 500 to 1000 unique species that have colonized the colon over the first year of life [48]; it works through a symbiotic relationship with the host, and having a role in metabolic, structural, and protective functions. 

However, these mechanisms could be disrupted due to altered gut microbial composition, referred to as “dysbiosis”. An enormous variety of disorders were linked to gut dysbiosis so far, including inflammatory bowel disease, intestinal infection, food allergies, asthma, diabetes, obesity, multiple sclerosis, autism, periodontitis and colorectal cancer [49]. Recent research has shown that significant changes in gut microbiota could promote the growth of otherwise low-harm bacteria that were responsible for disease, for instance, observed Enterobacterial bloom noticed during inflammation in gastrointestinal tract [49].

It was shown in in vivo studies that animals depleted of microbes had increased susceptibility to infection and serious defects of mucosal immune system [50]. Moreover, the microbiota is responsible for the immune system modulation by the regulation of inflammatory cytokines, plays a role in metabolic activity regulating the production of short chain fatty acids and influences significantly of host fat storage [51,52,53]. 

The gut microbiome is influenced by many factors, ranging from those related to the method of childbirth delivery (vaginal delivery versus cesarean section) and early infancy, e.g., infant feeding, through the further lifestyle and diet, including gastrointestinal infections and antimicrobial treatment [48,50,54]. Recent epidemiological and clinical genomics studies in humans, as well as in vitro and in vivo animal studies, have shown that gut microbial communities play a key role in the pathogenesis of gastrointestinal diseases.

It is noteworthy that the composition of the intestinal microbiota is dominated by anaerobic bacteria, which are 2–3 orders of magnitude more numerous than the facultative anaerobic and strict aerobic bacteria. It is estimated that about 1000 species occur simultaneously in one individual [55]. However, the total number of species inhabiting the intestines of humans is estimated at 35,000, as the result of a very high interindividual variability in the species composition of the intestinal microbiota [56]. As established by metagenomics sequencing, ten bacterial groups were found in the human gastrointestinal tract, of which *Firmicutes* and *Bacteroidetes* are the most important [57]. Similarly, Arumugam et al. divided human gut microbiota into three clusters, identified by their enrichment in *Bacteroides* (Enterotype 1), *Prevotella* (Enterotype 2) and *Ruminococcus* (Enterotype 3) [58]. 

**Figure 2:**
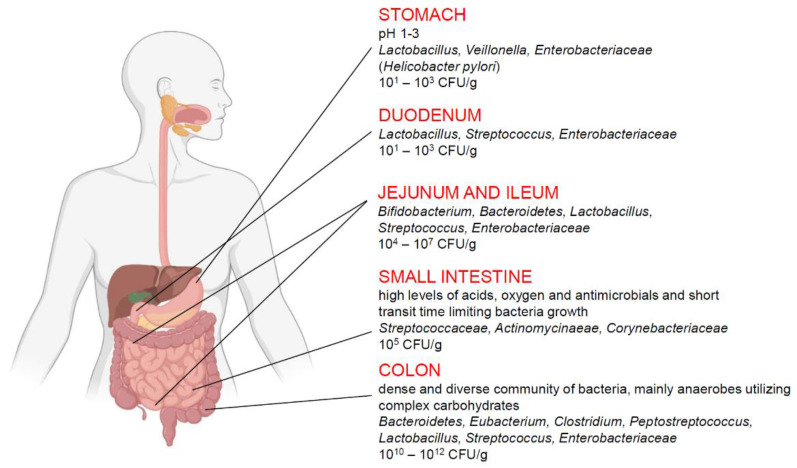
Composition of human intestinal microflora in the selected sections of the digestive tract. Colon microflora seems to have the greatest influence on the development of IBS.

The human intestinal microflora changes in different sections of the digestive tract (Figure 2). The smallest number of bacteria is found in the stomach (about 10^1^ bacteria/g), where the conditions for bacterial growth are the most difficult due to the very low pH. In the duodenum, the number of bacteria is estimated at 10^3^/g and increases gradually in each subsequent section of the intestine, reaching 10^12^/g in the colon [59]. In addition, a high variability in composition of the microflora from different parts of the gastrointestinal tract was reported [56]. The stomach is inhabited mainly by bacteria from the genera *Lactobacillus, Veillonella*, and *Helicobacter*, whereas the small intestine is colonized mainly by bacteria from the *Bacilli* and *Actinobacteria* classes with predominantly *Streptococcaceae*, and *Actinomycinaeae* as well as *Corynebacteriaceae* families, respectively. In contrast, the colon is dominated by the *Bacteroidetes* class and bacteria from the *Lechnospiraceae* family. Recent articles have highlighted that physiological gut microbiota variations as well as the specific enterotype, which are crucial in delicate host-microorganisms balance, and could determinate the development of some inflammatory intestinal disorders or significantly affect the course of the disease [60]. 

Importantly, the abundance and composition of the intestinal microbiota is not only associated with a section of the digestive tract but also with a sampling site, e.g., the surface of epithelium that is covered with a thick layer of mucus that separates it from the intestinal lumen. For instance, Swidsinski et al. [61] isolated *Clostridium, Lactobacillus* and *Enterococcus* genera from the epithelial layer and mucus of the small intestine, while the faeces of the studied subjects were enriched by additional *Enterobacteriaceae* as well as bacteria from *Streptococcus, Bacteroides* and *Bifidobacterium* genera.

It should be noted that bacteria are not the only prokaryotic inhabitants of the intestinal tract but coexist with another group of prokaryotic organisms, namely, Archaea. Although Archaea are not found in the intestines of all people [62], their ability to produce methane, so-called methanogenic archaea, makes them an important part of the intestinal microbiota in animal studies, methane has been shown to slow the passage of the small intestine, which may facilitate the development of constipation. The dominant archaeal species and methane producer in the human intestine is *Methanobrevibacter smithii.* Another methanogenic species is *Methanosphaera stadtmanae*, which has the most limited metabolism of all methanogenic archaea [63], explaining its lower occurrence than *M. smithii* [64,65]. Unlike *M. smithii*, which produces methane from hydrogen and carbon dioxide, *M. stadtmanae* uses methanol to produce methane and to synthesize ATP [63]. Remarkably, the level of methanol increases with pectin degradation by anaerobic bacteria, e.g., some species of *Bacteroides* [66], which enables *M. stadtmanae* to occur in the intestinal environment where many species of this type of bacteria are found.

It is believed that gut microbiota remains in a similar quantitative and qualitative composition over the time within individuals [67]. However, the influence of environmental factors as well as illnesses and drug use are able to change this ecosystem significantly. It hypothetically may lead to a number of disturbances, including IBS. Data published firstly in 2007 seemed to support this opinion; a meta-analysis of data taken from nine studies showed a strong association between gastrointestinal infection and IBS development (OR 5.9; CI: 3.6-9.5) [68]. The risk of IBS after an acute episode of gastroenteritis was associated with the young age of patients, its psychological disturbances and a less prolonged onset of the disease. Later published studies showed that IBS patients had a different microbiome to that of healthy controls; microbial diversity in the colon was significantly changed since several members of *Bacteroidetes* phylum were increased 12-fold in patients, while healthy controls had 35-fold more uncultured *Clostridia*. [69,70]. However, according to other researchers the role of microbiota is questioned since the criteria that we define as healthy microbiome is still unclear [6]. Moreover, the systematic review concerning gut microbiota in IBS did not answer the question, as to whether particular microbiota could be attributed to any subtype of IBS [71].

## 5. Small Intestinal Bacterial Overgrowth (SIBO)

Small intestinal bacterial overgrowth (SIBO) is a condition when there is an abnormal increase in the overall bacterial population in the small intestine (particularly types of bacteria not commonly found in that part of the digestive tract) together with a constellation of gastrointestinal symptoms. Its diagnosis is made on the basis of bacterial density (both aerobic and anaerobic): a threshold of >10^3^ colony forming units (cfu)/ml is recommended as a positive test result [72]. In some studies, a higher bacterial concentration was suggested (10^5^ cfu/ml) than what was based in traditional microbiological standards [7,73,74]. SIBO symptoms include abdominal pain, belching, bloating, diarrhea, distension, flatulence and indigestion; because of their broad spectrum in patients, they cannot be used to establish the diagnosis alone. Small bowel culture is generally accepted as the best diagnostic method [75], but it is invasive, expensive and has some methodological limitation [76]. On the other hand, breath testing is noninvasive and a safe diagnostic method for SIBO, however, its interpretation is complex and difficult in some cases [72]. There are several factors that predispose for SIBO: female sex, old age, use of proton pump inhibitors or opioids, dyspepsia, inflammatory bowel disease (IBD), IBS (irritable bowel syndrome), different types of abdominal surgery, diabetes and others [76]. 

Based on the observation that patients with both SIBO and IBS experienced similar symptoms, some researchers investigated the link between these two conditions. Firstly, epidemiological studies showed that the frequency of SIBO among IBS subjects ranged from 4% to 78% compared to healthy controls, of which only 1% to 40% had SIBO [77,78,79]. The others have postulated that SIBO is a potential cause of symptoms in IBS patients. The rationality of this hypothesis was based on the evidence that high numbers of coliforms were present in small bowel of IBS patients. Abnormal breath testing showing high prevalence of SIBO in IBS patients, and the observation that SIBO eradication caused significant relief of IBS symptoms further confirmed this phenomenon [80,81]. Additionally, it was found that SIBO occurred more often in IBS with diarrhea (35.5%) than in patients with IBS constipation subtype [79]. Therefore, this “SIBO hypothesis” has led to the use of antibiotics to treat IBS patients without constipation.

## 6. Antibiotics as a Treatment Options for IBS

The discovery of antibiotics, as any small molecule, produced by microbes, with antagonistic properties on the growth of other microbes, is considered one of the greatest discoveries of the 20th century. It is known that the way antibiotics work involved their interaction with bacteria, which affects bacterial survival in the mode of action that it is sufficiently potent to be effective against infection and simultaneously presents minimal toxicity, at therapeutic concentrations [82,83]. In the years 1940–1960, called the antibiotic golden age, the most antibiotic classes in use today were identified, which resulted in unparalleled reduction of infection disease risk in the world. The use of antibiotics for IBS came about when scientists noticed that there was a rationale for the benefit of such treatment, since bacteria are involved in the pathogenesis of IBS. Antibiotic elimination of the bacteria improves the clinical stage of subjects suffering from IBS, and that when the bacteria return, the symptoms return. On the basis of proof of a bacterial cause of IBS, antibiotics may be a good choice of therapy. Therefore, several controlled studies were performed to elucidate the efficacy of different antibiotics in IBS. It has been established that the ideal drug should meet the following conditions: broad-spectrum antibiotic with minimal side effects and low resistance. Firstly, absorbable antibiotics have been successfully tested; tetracycline, amoxicillin/clavulanate, metronidazole and norfloxacin significantly reduced the colonic overgrowth [84,85]. Since, these drugs may cause a serious side effects, more recent studies have demonstrated beneficial effects, especially with non-absorbable antibiotics that can selectively eradicate gut flora [86,87,88,89] (Table 1).

### 6.1. Neomycin

In a randomized, double-blind placebo-controlled trial of 111 IBS patients, recruited according to Rome I criteria, the efficacy of neomycin was compared to placebo [90]. In an intention-to-treat analysis, the authors showed that neomycin resulted in a 35.0% improvement in a composite score, compared with 11.4% for placebo (*p* < 0.05). Additionally, patients reported a percent bowel normalization of 35.3 after neomycin, compared with 13.9% for placebo (*p* < 0.001). Moreover, these effects were even stronger in 39 patients with constipation subtype of IBS; the global improvement was observed in 32.6% of neomycin treated IBS subjects versus 5% in control group (*p* < 0.001). However, neomycin treatment also has disadvantages: 25% of patients observed in the previously mentioned study, did not normalize their breath test abnormalities. Secondly, there is evidence that neomycin may produce rapid and durable clinical resistance, as shown in Yang’s study, wherein 75% of patients taking conventional antibiotics such as neomycin did not respond to subsequent therapy [86]. Therefore, the need for new antibiotic for IBS symptoms treatment has arisen with a key features of being non-absorbable, gut specific, with low bacterial resistance and very limited side effects and broad spectrum.

### 6.2. Rifaximin

The minimally absorbed antibiotic rifaximin, derivate of rifamycin, initially indicated for travelers’ diarrhea and hepatic encephalopathy, was extensively studied for the treatment of IBS [91,92,93,94]. The above studies have demonstrated that rifaximin treatment (800-1650mg daily) for 10 or 14 days caused the improvement in IBS symptoms and IBS-related bloating. Rifaximin clearly affects the composition of gut microbiota, however it has also been proposed that it may significantly influence microbiota functions such as adherence, virulence and metabolism or have direct anti-inflammatory actions [95]. The drug was studied in two randomized, double-blind, placebo trials, named TARGET 1 and 2, in which a total of 1260 IBS patients without constipation, according to Rome II criteria, were involved. When rifaximin was given at the dose of 550 mg three times a day for 2 weeks, it resulted in a significant relief of global IBS symptoms for at least 2 of the first 4 weeks after treatment (40.7% vs. 31.7% for placebo, pooled: *p* < 0.001). Moreover, a marked number of patients suffered IBS-associated bloating felt relieved from the symptoms (40.7% vs. 30.3% for placebo, pooled; *p* < 0.001). Most importantly, both improvements remained durable for the 10 weeks after cessation of the treatment [96].

A more recent randomized TARGET 3 trial (randomized, double-blind and placebo controlled) was performed to check the safety and efficacy of repeated rifaximin treatment. The authors recruited 636 IBS-D patients who previously had responded to the drug (given for 14 days), but they had symptoms recurrence. Next, they were given either rifaximin (550 three times daily) or a placebo for 2 weeks, then followed by 4 weeks drug free follow-up period. As reported in this study, patients treated with rifaximin showed statistically significant improvement: the percentage of responders for abdominal pain was 50.6 vs. 42.2% in placebo group. (*p* = 0.018) during at least 2 of 4 weeks of follow-up time [97]. The same authors mentioned that marked improvements were found for prevention of recurrence, durable response, and bowel movement urgency, while adverse event rates were low and similar between groups. However, it must be underlined that IBS patients who started repeated rifaximin treatment had lower symptom severity scores compared to their baseline before the first drug administration. The meta-analysis based on clinical outcomes of 1805 IBS subjects showed that rifaximin was considerably more efficacious than the placebo for global IBS symptom improvement (OR 1.57, CI: 1.22–2.01). Rifaximin treatment reduced the risk of IBS symptoms by 16% (RR 0.84, CI: 0.78–0.90) with a NNT (number need to treat) of 9 [10]. The other study was concentrated on safety and tolerability of the drug. The meta-analysis has shown that only one patient receiving rifaximin would stop the therapy because of the adverse effects for every 846 subjects who could benefit from the treatment; NNT = 10.6; number needed to harm (NNH) = 8971 [98]. Therefore, the American College of Gastroenterology has issued recommendations for rifaximin on the basis of data mentioned above. It certainly caused that the drug is FDA approved for the treatment of IBS with diarrhea. In 2018 rifaximin was included in the Polish recommendations for non-constipated IBS patients (IBS with diarrhea, IBS with mixed stool pattern, and IBS unclassified) to reduce general symptoms and the severity of flatulence and/or diarrhea. The recommended dose is 400 mg for times a day for 14 days [99]. The high degree of consensus among experts and the significant strength of evidence were emphasized. The beneficial results of rifaximin might indicate that the bacteria strain(s) responsible for development of IBS are more susceptible to its action compared to other strains that reside in the digestive tract. 

## 7. Do diet or Probiotics Matter?

Diet modification has long been gaining importance as an inseparable element in the treatment of many diseases. There are data showing that IBS patients, more frequently than healthy subjects, reported adverse reactions to meals; in particular their intolerance was mainly attributed to gluten, wheat, lactose and fermentable oligosaccharides, disaccharides and monosaccharides and polyols (FODMAP) [100]. Therefore, many doctors (and many patients by themselves) begin IBS treatment by changing the diet that triggers the symptoms. The most commonly used is fiber, although its non-soluble forms (as in bran) could even intensify abdominal pain and bloating in IBS subjects [101]. On the other hand, soluble fiber obtained from psyllium husk was beneficial for the treatment of IBS patients with constipation, as shown in meta-analysis in which total of 499 subjects were studied (relative risk of being symptomatic was 0.83, CI: 0.73–0.94) [10]. Moreover, recent data has shown that psyllium could significantly reduce the inulin-related gas formation in IBS patients [102]. The authors speculate that diet with adequate amounts of viscous fiber is able to increase tolerability of prebiotics in IBS patients (without exacerbating flatulence). 

Recently, there is a noticeable interest in dietary modification for IBS, however, the most promising results are related to fermentable oligosaccharides, disaccharides and monosaccharides and polyols contained in food. It is believed that stone fruits, legumes, lactose containing food, artificial sweeteners, which are very rich in FODMAP, may worsened conditions of IBS patients, because of fermentation and osmotic effects in colon [103]. Interestingly, the work of Halmos et al. showed in a randomized trial that low-FODMAP diet diminished global IBS symptoms scores; both bloating and abdominal pain were significantly reduced when compared to normal local diet [104]. Traditionally, the doctors’ dietary recommendations in IBS advised patients to eat small portions, regularly, and to avoid insoluble fibers, foods reach in fat, caffeine and alcohol. Subsequent research was aimed to compare this conventional dietary recommendation to low FODMAP diet; two randomized trials did not show any significant differences in overall response to IBS therapy between them [105,106]. Moreover, long-term reduction of intake of FODMAPs is able to significantly affect the intestinal microbiome composition [107] and the use of such a diet is not recommended over a long period of time. Recently, the popular abandonment of gluten in diet, especially in some dermatological and endocrine diseases or sliming, (which is beyond the official recommendations), also has no application in the treatment of IBS [108]. 

According to the arrangements of the consensus from 2014, probiotics are defined as “live microorganisms that, when administered in adequate amounts, confer a health benefit on the host” [109]. Despite the fact that their role has been recognized since 1908 [110], their exact way of influencing the human body is not known. The data showing that probiotics can inhibit pathogenic bacteria overgrowth and improve the gut barrier function or modulate the secretion short chain fatty acids and neurotransmitters [111], has awakened the hope they could be used in IBS treatment. However, recently published meta-analysis reported conflicting data; seven studies have proved the efficacy of probiotic supplementation on symptoms in IBS patients, while four other trials failed to do so [110]. Therefore, the ability to make the precise recommendation seems to be premature. However, different conclusions came out from the recently published systemic review, which had analyzed prospective randomized clinical trials studying 4321 IBS patients, published between 2000 and 2019 [112]. The authors indicated that products containing *Lactobacillus spp.* significantly reduced abdominal pain and flatulence score and improve QOL (quality of life) in these patients. Moreover, *Bifidobacterium* containing formulations could ameliorate stool urgency and other principal IBS symptoms. Important limitations of the study, as underlined, were the relatively short period of observation of 40% of the interventions and the poor methodology in some cases. Moreover, several side effects were reported, including abdominal pain, diarrhea, heartburn, nausea and flatulence; it was found difficult to distinguish whether they occurred due to probiotics or due to disease itself.

In order to relieve IBS symptoms, attempts are being made to use probiotics in conjunction with prebiotics. Such a combination is called synbiotics, and it is believed that it has a synergistic effect by inhibiting the growth of pathogenic bacteria and enhancing the number of beneficial organisms. Prebiotics are oligosaccharides, galactooligosaccharides, inulin, lactulose, resistant to the action of digestive enzymes in the gastrointestinal tract, which undergo bacterial fermentation and stimulate the growth of beneficial intestinal microflora. Silk et al. [93] demonstrated a beneficial effect of prebiotics administered to IBS patients. Compared with the placebo group of patients, in the group receiving a prebiotic (a mixture of galactooligosaccharides), the qualitative changes in the bacterial flora were observed in the stool (relative proportions *of Bifidobacterium spp*.), as well as a significant improvement in stool consistency and the reduction of complaints reported by the patients.

Recently published observations have focused attention on postbiotics, i.e., the bacterial free metabolites secreted by probiotics strains, as a better and safer strategy in IBS therapy, since they have beneficial immunomodulatory functions and a role in gut epithelium protection [113,114]. Moreover, data obtained by Perez et al. showed that *Lactobacillus plantarum* strains together with *Pediococcus acidilactici* have a positive clinical outcome in IBS, because this probiotic mix produced postbiotic molecules, including acetate and antimicrobial compounds, against IBS associated microorganisms [115]. However, further investigation is needed as to whether postbiotics and bioactive compounds may be an effective way to increase the potency of probiotics to turn them into functional ingredients or therapeutic agents in IBS.

## 8. Fecal Microbiota Transplantation

Preliminary data in IBS patients show a positive response to intestinal microflora transplant (FMT), which agrees with the concept that the composition of the gut microflora plays an important role in the pathogenesis of IBS. It is noteworthy that there is no consensus regarding FMT procedure, and different routes of administration (e.g., oral capsule, nasal infusion, and colonoscopy), formulations (e.g., frozen, dried and fresh), as well as the number and type of donors were studied. However, a recent meta-analysis of five randomized controlled trials in 267 IBS patients showed that colonoscopic FMT delivery was effective, while nasogastric tube delivery only tended to benefit, while oral capsules provided no benefit [116]. In another randomized and placebo-controlled study assessing the effectiveness of FMT via colonoscopy in patients 18-75 years of age with IBS with diarrhea or with diarrhea and constipation, clinically significant improvement in symptoms was observed after 3 months in 65% of patients compared to 43% of control subjects receiving their own stool [117]. Interestingly, better results were obtained in patients with frozen, not fresh, fecal microflora [117]. Likewise, randomized evaluation of the efficacy of intra-infusion FMT in 62 patients with refractory IBS (defined as failure of ≥3 conventional therapies), of all subtypes with predominant flatulence, was performed by El-Salhy et al. [118], and revealed improvement of both IBS symptoms and quality of life in 56% of the patients after 12 weeks of receiving FMT in comparison with 26% of the placebo receiving subjects (*p* = 0.03). In addition, 21% of patients who received FMT reported an improvement in symptoms for more than 1 year, compared with only 5% of patients who received placebo [118].

However, it must be emphasized that FMT is not free of potential adverse effects. For example, a study by El-Salhy et al. reported side effects in 20% of the FMT group versus 2% of the autologous FMT group, including two patients who developed diverticulitis in the FMT group and none with diverticulitis in the FMT group with autologous FMT [118]. Although most of the side effects associated with FMT are mild and self-limiting and serious side effects appear rare, further clinical trials are needed before considering this approach in clinical practice.

## 9. Conclusions

IBS is a very common disease with unclear pathophysiology involving genetic, physiological and psychological factors, which may be directly or indirectly connected with alterations in the composition of the gut microbiota. The latter contribute to the pathogenesis of IBS by affecting the gastrointestinal immune system, mucosal permeability, intestinal motility, visceral sensation, gut-brain communication, and intestinal fermentation. Therefore, therapy targeting the gut microbiota, e.g., based on pre-, pro- or postbiotics, may be a promising treatment for IBS. However, there are potential disadvantages to each of these treatments. For instance, prebiotics are often associated with undesirable abdominal symptoms and there is no strong evidence to support their effectiveness. In contrast, although the beneficial effects of probiotics on IBS symptoms have been reported by several studies, their heterogeneous methodology impedes drawing reliable conclusions. Thus, more research is required to determine the effectiveness of probiotics in terms of a probiotic type, dose, side effects, duration of treatment, and to identify suitable candidates. On the other hand, the recently developed drugs, especially non-systemic antibiotics such as rifaximin, have been successfully implemented in treating patients in the hope that they will have a significant impact on treating IBS symptoms without harming health in the long term. Likewise, various alternative treatment options, including fecal transplantation, are being explored and analyzed, but more evidence is needed from larger and well-controlled studies.

## Figures and Tables

**Table 1:** Efficiency of various antibiotics in IBS treatment.

Antibiotic	Efficiency in IBS treatment
Tetracycline, Amoxicilline clavulanate, Metronidazole, Norfloxacine	Moderate effect on IBS symptoms Possible systemic side effects Many cases of clinical resistance High risk of *Clostridioides difficile* infection
Neomycin	Rapid and durable clinical resistance Weak effect on IBS symptoms
Rifaximin	Effective in improvement of IBS symptoms including abdominal pain, bloating and diarrhea Weak effect on constipated patients Affects the composition of gut microbiota Direct anti-inflammatory actions Low possibility of clinical resistance or *Clostridioides difficile* infection

## Data Availability

No new data were created or analyzed in this study. Data sharing is not applicable to this article.

## Abbreviations

IBS	irritable bowel syndrome
CNS	central nervous system
IBD	inflammatory bowel disease
SIBO	small intestinal bacterial overgrowth
IBS-D	IBS with diarrhea
IBS-C	IBS with constipation
FODMAP	fermentable oligosaccharides, disaccharides and monosaccharides and polyols
FMT	fecal microbiota transplantation
QOL	quality of life
NNT	number need to treat
NNH	number needed to harm
ATP	Adenosine triphosphate
5-HT4	***5***-Hydroxytryptamine receptor 4
5-HT3	***5***-Hydroxytryptamine receptor 3
